# Evolution of Plant B Chromosome Enriched Sequences

**DOI:** 10.3390/genes9100515

**Published:** 2018-10-22

**Authors:** André Marques, Sonja Klemme, Andreas Houben

**Affiliations:** 1 Laboratory of Genetic Resources, Federal University of Alagoas, Av. Manoel Severino Barbosa, 57309-005 Arapiraca—AL, Brazil; 2 Biology Centre, Czech Academy of Sciences, Institute of Plant Molecular Biology, Branišovská 31, CZ-37005 České Budějovice, Czech Republic; sonja.klemme@gmx.de; 3 Leibniz Institute of Plant Genetics and Crop Plant Research (IPK), Corrensstrasse 3, 06466 Gatersleben, Germany; houben@ipk-gatersleben.de

**Keywords:** B chromosome, satellite DNA, mobile element, organelle DNA, chromosome evolution

## Abstract

B chromosomes are supernumerary chromosomes found in addition to the normal standard chromosomes (A chromosomes). B chromosomes are well known to accumulate several distinct types of repeated DNA elements. Although the evolution of B chromosomes has been the subject of numerous studies, the mechanisms of accumulation and evolution of repetitive sequences are not fully understood. Recently, new genomic approaches have shed light on the origin and accumulation of different classes of repetitive sequences in the process of B chromosome formation and evolution. Here we discuss the impact of repetitive sequences accumulation on the evolution of plant B chromosomes.

## 1. Introduction

Supernumerary B chromosomes (Bs) are not required for the normal development of organisms and are assumed to represent a specific type of selfish genetic elements. As a result, Bs follow their own species-specific evolutionary pathways. Several recent studies have confirmed the widely accepted view that Bs are derived from their respective A chromosome (A) complement [1,2,3,4,5,6]. Although Bs may vary in structure and chromatin properties in a species-specific way, their de novo formation is probably a rare event, because the occurrence of similar B chromosome variants within related species suggests that they arose from a single origin either from the same or from a related species [7,8].

The evolution of B chromosomes’ (Bs) architecture has historically been of interest mainly at the cytogenetic level, with a recent focus on more molecular and genomic level studies (reviewed in [9,10,11]). Here we focus our review on the impact of repetitive DNA on the evolution of plant B chromosomes.

## 2. Methods and Tools to Characterize the High-Copy DNA Composition of B Chromosomes—Past and Future

First analyses of the DNA composition of B chromosomes were based on renaturation kinetics and gradient density centrifugation. These approaches showed that in rye the heterogeneity of repeats and ratio in Bs did not differ from As [12]. In maize the buoyant densities of DNA from plants with and without Bs were found to be alike [13]. Later, the use of comparative restriction endonuclease digestion of genomic DNA with and without B chromosomes of several *Glossina* species was introduced to characterize Bs. [14] Next generation sequencing (NGS), in combination with bioinformatics, led to an advance in our understanding of DNA sequence composition, functional gene content, and evolution of eukaryotic B chromosomes. In principle, NGS-based methods for the identification of B-specific sequences can be classified in two strategies [10]. In the first of these, DNA reads of microdissected or flow-sorted B chromosomes are used. Chromosome flow sorting usually produces larger amounts of DNA than microdissection. The most significant disadvantages of sequencing microdissected probes are the low amount of template DNA, contamination by surrounding material, and PCR amplification bias. Consequently, in silico purification of produced sequence reads is recommended. However, recent advances in single cell analysis demonstrated that even the DNA of a single haploid nucleus is sufficient for NGS analysis [15].

The second strategy requires sequencing of two whole genome datasets of the same species, one containing Bs (+B) and one without Bs (0B). It is an indirect approach because B-derived sequences are compared against the 0B-derived sequences as an additional step. This approach identifies B-candidate sequences where the ratio of aligned sequences is significantly increased in the +B dataset compared to the 0B dataset.

Identification of B chromosome-enriched sequences, like satellite repeats, mobile elements or organelle-derived sequences by similarity-based clustering of next generation sequence reads was achieved using the RepeatExplorer software [16,17] for rye [1] and *Plantago lagopus* [18]. With the RepeatExplorer and RepeatMasker programs the satellite DNA composition of the migratory locust (*Eyprepocnemis plorans*) B chromosomes was determined [19]. The ‘satellitome’ in the grasshopper *Eyprepocnemis monticola* consists of 27 satellite DNAs [6], less than half of the migratory locust, where 62 were found [19].

To identify repetitive elements or their parts the RepeatExplorer software [20] uses graph representation of read similarities to identify sequence clusters of frequently overlapping sequence reads [21]. Also, this software provides information about repeat quantities and others. The repeats are annotated based on BLASTN and BLASTX similarity searches to custom databases of repetitive elements and repeat-encoded conserved protein domains.

The in silico ‘coverage ratio analysis’ relies on a read alignment analysis performed for each of the 0B and +B datasets that subsequently are investigated for differences in the read coverage ratio. The strategy was used to determine the B chromosome sequence content of the cichlid *Astatotilapia latifasciata* [3]. Coverage ratio analysis revealed that the B chromosome contains thousands of sequences that have been duplicated from almost all standard chromosomes of this species, although most B-located genes are not contiguous. Subsequent sequence analysis of microdissected *A. latifasciata* Bs confirmed this conclusion.

An additional comparative approach is the *k*-mer based analysis termed ‘*k*-mer frequency ratio analysis’. Similar to coverage ratio analysis, it allows the analysis of 0B and +B sequence sets and investigates the differences in the *k*-mer frequency ratio. The web-based tool Kmasker [20] can be applied to run a *k*-mer frequency ratio analysis. The advantages and disadvantages of the different strategies are further discussed in Ruban, Schmutzer, Scholz and Houben [10].

Mention of specific programs in this review denotes only previous use in cited studies and does not imply endorsement.

## 3. Accumulation of B-Specific Repeats

Because of the high degree of evolutionary conservation and the high copy number, 5S and 45S ribosomal DNA (rDNA) satellite repeats have often been used as in situ hybridization probes for the analysis of Bs. The involvement of rDNA satellites in the evolution of plant Bs does not appear to be accidental, because rDNA loci have been detected on Bs of many species of plants (e.g., *Crepis capillaris* [22]), *Brachycome dichromosomatica* [23,24], *Aegilops* [25], and animals (e.g., *Haplochromis obliquidens* [26]) and *E. plorans* [27]. Although transcription of B chromosome-located rDNA was long believed not to occur [28], more recent studies have shown that indeed they are expressed, for instance the B-located 45S rRNA genes of the plant *C. capillaris* [29] and the grasshopper *E. plorans* [30]. In contrast, the ribosomal RNA genes specific to the B chromosomes in *B. dichromosomatica* are not transcribed [23]. Differences in posttranslational histone modifications, such as acetylation or methylation of histone, between A and B chromosomes, have been demonstrated [31,32,33,34,35]. Another possibility is that suppression of genes may occur due to nucleolar dominance such that the rRNA genes on the A chromosomes are active at the expense of B chromosome-located rRNA genes [24]. The inactivity of B chromosome rDNA could explain the presence of multiple ITS (internal transcribed spacer) sequences, since homogenization of rDNA spacers is thought to occur in transcribed regions only. Concerted evolution is a typical feature of the rDNA [36], but the mechanisms that control it may not include non-transcribed rDNA regions [37,38]. Since no homogenization occurs between the rDNA of A and B chromosomes, and since Bs are less active, one might expect further sequence erosion of B-located sequences. For the B chromosome-like paternal sex ratio chromosome of the wasp *Trichogramma kaykai,* it has been postulated that an increase in the number of different members of ITS sequences could be the evolutionary consequence [39].

In the herb *P. lagopus* L., the B chromosome is the product of a spontaneous amplification process of 5S ribosomal DNA derived repeats [18,40]. Interestingly, the amplification of satellite DNA has been used for the formation of engineered mammalian chromosomes (‘satellite-DNA-based-artificial-chromosomes’ [41]). In contrast to the situation with animals, the molecular mechanism of sequence amplification in plants is poorly understood. However, except for tobacco [42], no amplification-stimulating DNA elements from plants have been identified thus far. Alternatively, B chromosomal rDNA sites could be a consequence of the reported mobile nature of rDNA [43]. Bs may be the preferred “landing sites” because of the relative inactivity of constituent sequences and independence from selective forces on the As. Increasing evidence also indicates that rDNA can change position within the genome without corresponding changes in the surrounding sequences [44,45,46]. Beside rDNA, Bs accumulate chromosome specific satellite DNA (satDNA) which are listed below according to species.

### 3.1. Rye

The first plant B chromosome-specific satellite repeat has been identified in rye. Comparative restriction digestion of 0B and +B genomic DNA resulted in the isolation of the high-copy repeats E3900 and D1100, which seem to have de novo evolved on rye Bs [47,48,49]. Both repeats have classical features of satDNA such as being tandemly repeated, although atypical features such as transcriptional activity and euchromatic histone modifications have been reported for these repeats [7,35]. They also show an atypical repeat unit size with 1.1 and 3.9 kb, for D1100 and E3900, respectively [47,48]. These two repeats seem to have been assembled from fragments of a variety of sequence elements [49]. Both contain fragments of mobile elements most likely generated by chromosomal rearrangements, although no coding sequence responsible for the autonomous mobility has been found [49].

**Figure 1:**
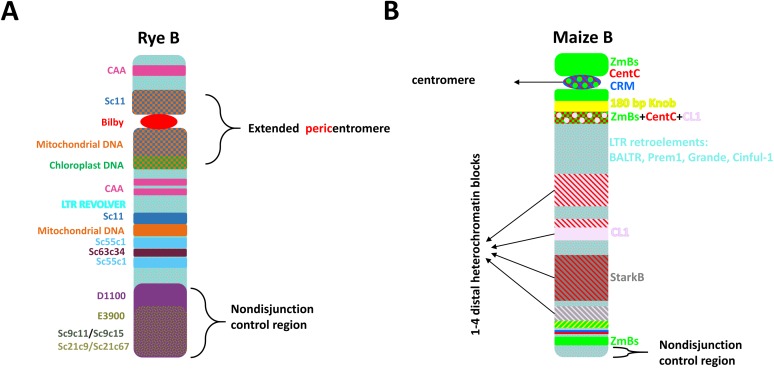
Model for the distribution of (**A**) rye and (**B**) maize B chromosome-enriched sequences. In both rye Bs and maize Bs only the very terminal region of B long arm is required for non-disjunction control [51,53,54].

NGS studies on rye Bs have significantly increased our knowledge about B-specific repeat accumulation and evolution [1,7,50,51]. Since the sequencing of rye B, several B-specific repeats have been identified, mostly being satDNA [1,52]. Furthermore, these studies have shown that rye Bs are descended from rearrangements of the rye standard A chromosomes 3RS and 7R, with subsequent accumulation of repeats and genic fragments from other A chromosomal regions, as well as insertions of organellar DNA [1]. In silico identification of the high-copy sequence fraction revealed several B-specific repeats [52]. An accumulation of B chromosome-enriched tandem repeats was found mostly in the nondisjunction control region of the B. This unique region is late-replicating and transcriptionally active. All B-enriched repeats are not unique to the B chromosome but are also present in other species of the genus *Secale* [52]. Moreover, while it was shown that Bs contain a similar proportion of repeats to the A chromosomes in regards to their total DNA content, the two differed significantly in composition. This was due to the accumulation of B-specific satellite repeats, mostly in the nondisjunction control region at the terminal part of the long arm, as well as in the extended pericentromere [1,7,52]. The high-copy composition of the rye B seems even more conserved than that of the A chromosomes as different hybridization patterns were found for the repeats ScCl11, Sc36c82 and Sc55c1 on As of different accessions but not on Bs [7], suggesting an important role for the maintenance of the B typical structure. An overall scheme of the rye B repeat composition is shown in Figure 1A.

### 3.2. Maize

The maize (*Zea mays*) B is also well studied and several works have been conducted aiming to understand its composition and accumulation mechanisms. Similar to rye Bs, maize Bs are also characterized by chromosome-type specific repeats [55,56,57,58,59]. The maize B centromere region contains a repetitive element ZmBs that is not present on the A chromosomes, which also shares homology over 90 bp with the maize knob sequence [55]. Later an approximately 700-kb domain that consists of all three previously described repeats (ZmBs repeat, CentC, and CRM) was described that is localised at the core centromere of the maize B and show enhanced association with CENH3 [60,61].

Two of the elements that are specific to the maize B are organized in long tandem arrays with repeat units of similar size. The ZmBs repeat, with approx. 1400 bp of unit length, is located in and around the B centromere as well as near the tip of the B long arm [55,56]. The CL-1 repeat, with approx. 1500 bp of unit length, is present in the first three heterochromatic blocks of the long arm [58,59]. Neither repeat has homology to any known open reading frames or sequences located on As. However, transposition of a retrotransposon and a Miniature Inverted-repeat Transposable Element (MITE) element involved in the genesis of the CL-1 repeat was detected [59] (Figure 1B).

Another B-specific repeat found on maize Bs is the Stark B element, which like D1100 and E3900 rye B elements is a retrotransposon-derived sequence. This repeat was formerly identified as B-specific sequence family with a relationship to the Prem1 family of maize retroelements, which are preferentially transcribed in pollen [57]. It is composed of repetitive sequences known from the A genome as well as novel sequences unique to the B. The StarkB element is much larger than the other B-specific elements of maize. StarkB copies vary by small insertions, deletions, and duplications as well as single-nucleotide polymorphisms. The minimum age of the StarkB repeat array was estimated to be at least 2 million years [56]. The formation of StarkB reflects a process that generates large amounts of DNA on the B chromosome. StarkB is also transcriptionally active. Therefore, the process that contributed to formation of the maize B combined pre-existing coding regions to produce novel transcripts [56].

StarkB is specifically located on heterochromatic domains, distributed throughout the third and fourth blocks of heterochromatin. Although this repeat is retrotransposon-derived it lacks the autonomous domains being characterized as non-autonomous chimeric element. StarkB is not arranged in arrays in contrast the maize B-repeats ZmBs and CL-1. Because the two blocks of heterochromatin that contain StarkB have persisted over many generations, their presence may play a role in B chromosome transmission [56].

### 3.3. *Brachycome dichromosomatica*

The daisy *B. dichromosomatica* has Bs of two different types, the larger Bs are somatically stable whereas the smaller, or micro Bs are somatically unstable. Both types of Bs contain clusters of 45S rDNA.

The large B carries a B-specific tandem repeat (Bd49) that is located mainly at the centromere [62,63]. Multiple copies of sequences related to this repeat are present on the A chromosomes of related species without Bs, whereas only a few copies exist in the As of *B. dichromosomatica* [63]. An isolated Bd49 clone was composed entirely of a tandem array of the repeat unit. However, in other clones the Bd49 repeats were linked to, or interspersed with, sequences that were repetitious and distributed elsewhere on the A and B chromosomes. One such repetitious flanking sequence had similarity to retrotransposon-like sequences and a second was similar to chloroplast DNA [64].

The micro Bs share DNA sequences with the As and the larger Bs, and they also have B-specific repeats (Bdm29 and Bdm54) [65,66]. Bdm29 is highly methylated and after in situ hybridization labelled the entire micro Bs. The Bdm29 is AT-rich with an insert of 290 bp long containing no significant subrepeats. A high number of Bdm29-like sequences was also found in the larger Bs of *B. dichromosomatica* and in other Bs within the genus, suggesting that the Bdm29 sequence is highly conserved and widespread [65]. The Bdm54 repeat is AT-rich with an insert of 477 bp long containing four copies of a subrepeat unit (TCGAAAAGTTCGAAG) as well as three perfect and four degenerate copies of a second short repeat (AGTTCGAA) that are embedded in the first unit [66]. Some micro B-located repeats have been shown to occur as clusters on the A chromosomes in a proportion of individuals within a population [67]. The observation that the genomic organization of the micro B is unlike anything found on the A chromosomes precludes their origin by simple excision from an A chromosome and also indicates that micro Bs do not integrate directly into the A complement to form polymorphic heterochromatic segments [66].

### 3.4. *Aegilops speltoides*

The *Aegilops* Bs are also known to accumulate several repetitive sequences being characterized by a number of A chromosome-localized repeats like Spelt1, pSc119.2 tandem repeats, 5S rDNA and Ty3-gypsy retroelements [26,68,69,70], as well as organelle-derived DNA [71]. However, no B-specific repeat has been found in this species yet.

### 3.5. *Plantago lagopus*

In *P. lagopus*, a weed of the Mediterranean region, a B-specific satellite PLsatB was identified that originated from sequence amplification including 5S rDNA fragments [41]. The satellite repeat PLsatB makes up 3.3% of the 1B genotype but only 0.09% of the 0B genotype [18]. In situ hybridization with the B-repeat revealed an almost uniform labelling of the Bs at meiotic metaphase I, while extended mitotic prometaphase chromosomes showed a more clustered distribution of the hybridization signals. In any case, no signals were detectable on the A chromosomes. Although PLsatB has evolved from 5S rDNA sequences, a 5S rDNA probe was not sufficient to label the Bs by fluorescence in situ hybridization (FISH) [18].

### 3.6. *Cestrum*

Species of *Cestrum* have shown large diversity in the accumulation and distribution of repetitive DNA families [72], and Bs have been described in six species and one interspecific hybrid: *C. strigilatum*, *C. diurnum*, *C. parqui × C. aurantiacum*, *C. intermedium*, *C. parqui, C. euanthes* and *C. nocturnum* [73,74,75,76]. Some of these repeats have already been identified and associated with B chromosomes [73]. In the hybrid *C. parqui × C. aurantiacum*, for instance, the B chromosome contains 35S and 5S rDNA and SSR AT-rich motifs [75]. Sequences of rDNA were also identified in Bs of *C. parqui*, *C. euanthes* and *C. nocturnum* [76]. In *C. intermedium* and *C. strigilatum*, besides C-Giemsa+/CMA+/DAPI+ bands [73], the Bs also display hybridization signals with the Gypsy-like retrotransposon probe but not with rDNA probes [73]. Some types of repetitive DNA were identified in A and B chromosomes in *C. strigilatum* and in species of this plant group, such as AT-rich SSR, 35S and 5S rDNA, C-Giemsa and C-CMA/DAPI bands and retrotransposons [77].

### 3.7. *Crepis capillaris*

In *C. capillaris* in situ hybridization of cells with labelled DNA derived from microdissected Bs confirmed that the B is composed mainly of sequences also present in the A chromosomes, but lacks the main repeats located on A chromosomes [78,79]. No B-specific repeat has been found. The highly abundant repeat B134 shows repeating units with a sequence similarity range from 69% to 90% and characterized by its richness in (CA)_n_ repeats. Members of this family are dispersed throughout the A and B chromosomes but are more concentrated in the pericentromeric heterochromatin of the B, indicating that the molecular organization of B heterochromatin is different from that of the As. B-located B134 repeats also have diverged from those on the As [79].

## 4. B Chromosome-Specific Accumulation of Organelle DNA

Mitochondrial and chloroplast DNA sequences are frequently transferred into the nuclear genome. This transfer usually dependents on recombination-based insertions of organellar DNA into the nucleus. As a result nuclear insertions of plastid DNA (NUPTs) or nuclear insertions of mitochondrial DNA (NUMTs) occur [80]. Nuclear transfer of organelle DNA is a well-known process (reviewed in [81,82,83,84]. Nuclear insertions NUPTs and NUMTs have been shown to be involved in the formation of new nuclear genes [83].

Large insertions of organelle DNA were found on the rye B chromosomes [1]. While plastid DNA is not absent from the As, the B localized sequences are considerably larger. They most likely stem from several independent insertion events. However, the evolutionary forces that resulted in organelle sequences organized as clusters on Bs are not well understood. Between Bs from different geographical origin there is no difference in organelle DNA content or distribution except for a pericentric inversion detected by FISH [7]. In contrast, the B chromosomes of *Ae. speltoides* of different accessions showed differences in abundance and location of organelle DNA [70]. However, all tested accessions did show accumulation in the B chromosomes over A chromosomes. When comparing A- and B-derived organelle reads to the original organelle-genome sequence of wheat, the reads from the rye B show less similarity to the source genome [1]. The inserts on the B therefore accumulated more mutations than A located inserts. It is also noticeable that the strongest accumulation happened in the pericentromere for rye Bs, and in the distal chromosome arms for *Ae. speltoides*. Pericentric insertions of organelle DNA have also been shown in rice A chromosomes [85]. Pericentromeric regions generally contain few functional genes, and this low gene density may facilitate the repeated integration of the organelle derived DNA [85]. Alternatively, consistent with the rapid evolution of centromeres [86] after sequence integration, subsequent amplification of these sequences might have occurred within this region. The confinement to mostly one region hints at two different possibilities for enrichment in mitochondrial DNA: (i) directed repeated insertion into the surroundings of this area, or (ii) few insertions with subsequent local amplification of these sequences. Although the second explanation seems more likely, the diverse nature of the organelle sequence reads suggests many independent insertion events instead of many copies derived from one incorporation. Interestingly it has also been shown that organelle DNA often integrates several fragments into one location [83]. This would fit well with data indicating many events rather than amplification of just one insertion [1].

What mechanism could account for the accumulation of organellar DNA in B chromosomes? It has been demonstrated that environmental stresses increase the incorporation of organelle DNA into the nucleus [84]. As the Bs, especially in higher numbers, can be considered as stress factors to the cell [87], their presence might increase the basal rate of DNA transfer. Transfer of organellar DNA to the nucleus is very frequent [81,88,89], but most of the “promiscuous” DNA is also rapidly lost again via a counterbalancing removal process [90]. If this expulsion mechanism is impaired in B chromosomes, then the high turnover rates that prevent such sequences on the A chromosomes from accumulating and degrading would be absent and allow for sequence decay. Thus, the dynamic equilibrium between frequent integration and rapid elimination of organellar DNA could be imbalanced for B chromosomes. This hypothesis is supported by higher divergence of B-derived NUMT reads compared to A-derived NUMT reads in *Secale cereale* [1]. Future analyses of other B-bearing species are needed to address the question as to whether organelle-to-nucleus DNA transfer is an important mechanism that drives the evolution of B chromosomes.

Another possibility for the accumulation is the dependence on double strand breaks (DSBs). If B chromosomes are more prone to DSB, this could aid in the insertion of random available DNA [71]. Organelle DNA on Bs might be underreported in genomic studies, since organelle DNA is generally filtered out during sequence analysis, due to contamination with DNA extracted from organelles. The presence of large insertions of organelle DNA on B chromosomes might be a plant specific phenomenon, as there have not been any reports of animal B chromosomes with mitochondrial insertions.

## 5. B Chromosome-Specific Accumulation of Transposable Elements

Recent works have demonstrated that B chromosomes accumulate DNA from various sources existing as amalgamations of mixed repeats and single-copy regions. In such a scenario, Bs would provide a safe haven for the accumulation and spread of transposable elements (TEs). This has been suggested as a mechanism through which some of the variability in mammalian Y chromosomes has arisen, as random insertions of transposable DNA into different regions of the Y chromosome would result in elements differing with respect to DNA composition and structure [80,91].

Although TEs compose the vast majority of repetitive DNA in eukaryotic genomes, very few studies have been conducted on plants carrying Bs. Thus, little is known about TE accumulation mechanisms on Bs. Evidence that Bs can provide an ideal target for transposition of TEs comes from works on the retrotransposon NATE (*Nasonia* Transposable Element) which has been described from the PSR (paternal sex-ratio) element of *Nasonia vitripennis* [92,93]. B-specific accumulation of Ty3/gypsy retrotransposons has been also reported for the fish *Alburnus alburnus* (L.) [94]. In plants, the rye Bs show B-specific accumulation of LTR (long terminal repeat) families [1,52]. In maize B several members of LTR retroelements were found showing differential accumulation and hybridization pattern compared to the As [56,61]. A retrotransposon has also been invoked in the transposition of chloroplast DNA into the repeat element Bd49 of the B chromosomes of *B. dichromosomatica* [64]. Thus, insertion of such elements may be responsible for the generation of structural variability in Bs [95].

### 5.1. Rye

Although most repeats are similarly distributed along As and Bs of rye, several transposons are either amplified or depleted on the B chromosome. For instance, the ancient retroelement Sabrina, abundant in all Triticeae and transcriptionally inactive in rye [96], is highly accumulated on As but less abundant on Bs. In contrast, the active element Revolver, as well as the predicted Copia transposon Sc36c82 seem to be more amplified on the Bs [52]. The B-specific gain of active mobile elements might have its cause in the lack of selection pressure on B chromsomes. Meiotic crossing-over has been proposed to remove mobile elements [97]. The B chromosomes of rye pair frequently with each other and themselves in meiosis [98], but the bivalents of Bs are often less connected by chiasmata than the As [99]. As proposed for plant Y chromosomes [100,101], reduced crossing-over might facilitate the accumulation of retroelements on Bs.

Based on these findings Klemme, Banaei-Moghaddam, Macas, Wicker, Novak and Houben [52] suggested a model for the selective accumulation of TEs on the B chromosomes of rye: “In the Triticeae ancestor, Sabrina was transposing and spread over the entire genome. After inactivation of Sabrina [96] before or during speciation of rye, the B was formed from the As with Sabrina still present. The newly evolving elements such as Revolver then became active and transposed throughout the rye genome. The dispensable nature of the B and the lack of selective pressure allowed for stronger accumulation of Revolver on the B, even further diluting the remnants of inactive elements which can no longer increase copy number.”

Additionally, other retroelements have been also found to be accumulated on rye Bs, for instance, the Copia elements Sc11c32 and Sc11c927, similar to the centromeric sequences Bilby and Sc11 (Figure 1A), expanded more in the extended pericentromere of the B than in those of As [51]. The presumed ancestral elements are still detectable in subterminal positions on As [7,52].

### 5.2. Maize

Furthermore, the maize B is also known to share the same centromere-specific retrotransposons (CRM elements) with the As [60]. Another retroelement called BALTR1 (B and A LTR element 1) was also found to be hybridized throughout the A and B genome, but showed enrichment near centromeres and on the B long arm [56]. The BALTR1 element was 7892 bp in length with LTR that were 1159 and 1173 bp, and possessed the internal coding sequences and the LTRs. The LTR of BALTR1 did not show similarity to sequences in public databases. Because the coding region was most similar to the CRM family of retrotransposons, this novel retrotransposon is likely a member of the Ty3/gypsy class [56]. Additionally, several other members of retroelements of LTR Ty3/Gypsy class were found on the maize Bs. For instance while both Prem1 and Cinful-1 elements showed hybridization signals more intense than the As with a similar distribution pattern as the centromere diffuse elements, the Huck element only hybridized weakly to the euchromatic region on the B [61]. The maize B is also uniformly labelled with the Grande element at the same level as the As [102]. An overview of maize B-enriched repeats is shown in Figure 1B.

## 6. Rapid Evolution of Repeats on Bs

The accumulation of repeats accompanies the evolution of Bs in several plant species, suggesting that the Bs most likely represents a suitable chromosomal context for satellite expansion. But why do Bs often accumulate repeats? 

There are at least two possible pathways for Bs to undergo repeat accumulation. One alternative source for the accumulation of repeats on Bs comes from studies conducted on rye where a restriction of meiotic recombination in Bs was observed, which showed variation in the frequency of bivalents formation among different genotypes [103,104]. This restriction of recombination can be considered as starting point for the independent evolution of Bs. The presence of fast-evolving repetitive sequences, could predispose a nascent B to undergo further rapid structural modifications required to establish and amplify new B-specific repeats.

A second alternative is that Bs are under reduced selective pressure due to their non-essential nature, as far as it does not affect its accumulation mechanism. Such chromosomal environment may be considered as a safe haven for non-coding fast-evolving sequences, such as promiscuous DNA as satDNA, organelle DNA and TEs, which are frequent genome hitchhikers able to settle in non-recombining regions. Thus, taking in account the features of Bs, these two pathways may explain the great diversification of repeats found on Bs.

Shrinkage and expansion of the Y chromosome is influenced by the sex-specific regulation of repetitive DNA. It was suggested that the dynamics of Y chromosome evolution is an interplay of genetic and epigenetic processes [80]. Many eukaryotic genomes contain a large proportion of repetitive DNA sequences. These sequences often colonize specialist chromosomes (Y, W or B chromosomes). In particular, the non-recombining regions of the Y chromosome, are subject to different evolutionary forces compared with autosomes. Repetitive DNA sequences often accumulate in the non-recombining regions of the Y chromosome [105]. A similar mechanism may take place in Bs, since it is known that, at least for rye, the frequency of meiotic recombination and proper bivalent formation may vary greatly among different genotypes as discussed above.

Because Bs are assumed to be non-essential they are also presumed to be evolutionarily neutral in host genomes and, thus, it is expected that high B sequence variability will be observed among different samples. The origin of B structural variants is most likely from a monophyletic origin from a unique type of ancestral B chromosome which afterwards diverged in different types through generations [7,8,106]. Indeed, there are several cases of B polymorphisms whether numerical or structural [107,108]. For the B chromosome of the grasshopper *E. plorans,* a large variety of structural variants has been demonstrated among many populations [107]. In plants, B polymorphisms have been mainly attributed to numerical polymorphisms [108,109]. Although, in a few cases B structural variants in natural populations have been identified e.g., *B. dichromosomatica* [106], *Ae. speltoides* [71] and *S. autumnalis* [110].

## 7. Evolutionary Aspects of Repeat Accumulation

The B-specific repeats E3900 and D1100 which are located in the non-disjunction controlling region of the rye B are characterized by unusual properties including a predisposition to instability [49]. There are similarities between StarkB element of maize and E3900. Both elements share sequences with the respective A genome and are composed of a complex mixture of unique sequences. Also, both are found in clusters intermixed with other B-specific sequences near the end of the chromosome [49,56]. These common features may display similarities in how these elements evolved. It has been suggested that alterations to the E3900/D1100 region of rye could influence the degree of chromatin packaging and affect the efficiency of B chromosome drive [49]. When the number of Bs in a population is too high, so that the fitness of the host is adversely affected, B variants with lower transmission would be selected [56]. Indeed, rye genotypes with different B chromosome transmission rates exist [103,104].

**Figure 2:**
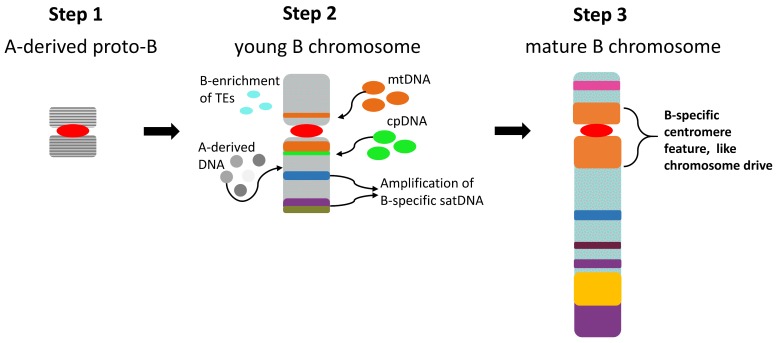
Model for the repeat accumulation and evolution of plant B chromosomes. (**1**) Proto-B chromosome derived as a result of multiple translocations and duplications of A chromosome fragments (harboring a functional centromere). (**2**) Gene erosion/silencing followed by restriction of meiotic recombination triggers B-specific repeat accumulation. (**3**) Mature Bs could achieve a high degree of B-specific repeats, an efficient drive mechanism and a tolerable impact on the fitness of the host organism. mtDNA: mitochondrial DNA; cpDNA: chloroplast DNA; satDNA: satellite DNA.

As a general model for the accumulation and evolution of repeats on plant Bs, we propose the following: (1) A proto-B chromosome was derived as a result of multiple translocations and duplications of A chromosome fragments and, therefore, shows a similar sequence composition as the original A chromosome fragments. The presence of a chromosome fragment processing a functional centromere to assure mitotic and meiotic segregation is essential. (2) Gene silencing/erosion followed by restriction of meiotic recombination and reduced selective pressure triggers a B-specific repeat accumulation due to the non-essential nature of the B chromosome. At this point the formation of a B chromosome-specific drive/accumulation mechanism is essential for the survival of the B chromosome. (3) Mature Bs are characterized by B-specific repeats and a tolerable impact of the Bs on the fitness of the host organism. Unless the B is eliminated, it could constantly accumulate sequences and change its structure (neutral mutations) and enhance its drive mechanism (positive selection) along its evolution. Furthermore, it is important to notice that the B centromeres at least in rye and maize show specific features to assure chromosome drive via nondisjunction at first or second pollen grain mitosis, respectively (reviewed in Houben [111]). Thus, the evolution of B-specific (peri)centromeric properties seems to be a key step in the evolution of Bs. Figure 2 shows a diagrammatic summary for the proposed model of repeat accumulation and evolution of Bs.

## 8. Concluding Remarks and Future Perspectives

As discussed above, Bs are expected to undergo reduced selective pressure due to their non-essential nature and, thus, exhibit larger variation than A chromosomes. However, it is remarkable to see that in fact some Bs have a relatively conserved structure with rare occurrence of structural variants across plant species [7,106]. In some cases, it is remarkable to see that the variation in the As might be even higher than that observed in the Bs, as for instance in *B. dichromosomatica* [106] and for the B-specific repeat ScCl11 in rye [7]. This suggests the existence of a control mechanism for the maintenance of a standard B structure. Although Bs seem always to have an A-derived architecture, they are frequently found to have accumulated several B-specific repeats and, at least in some cases, insertions of organellar DNA. Furthermore, they may show notable variation in their quantitative repeat composition. These unique features observed on Bs highlight on the one hand the origin of Bs from As, and on the other a different evolutionary pathway of As and Bs. It seems that the B acts like a “genomic sponge” which collects and maintains sequences of diverse origins.
