# Correction: Marques, A. et al. Evolution of Plant B Chromosome Enriched Sequences. *Genes* 2018, *9*, 515

**DOI:** 10.3390/genes10020085

**Published:** 2019-01-26

**Authors:** André Marques, Sonja Klemme, Andreas Houben

**Affiliations:** 1 Laboratory of Genetic Resources, Federal University of Alagoas, Av. Manoel Severino Barbosa, 57309-005 Arapiraca—AL, Brazil; 2 Biology Centre, Czech Academy of Sciences, Institute of Plant Molecular Biology, Branišovská 31, CZ-37005 České Budějovice, Czech Republic; sonja.klemme@gmx.de; 3 Leibniz Institute of Plant Genetics and Crop Plant Research (IPK), Corrensstrasse 3, 06466 Gatersleben, Germany; houben@ipk-gatersleben.de

The authors wish to make the following modification in their review [1]. Change *Eyprepocnemis plorans* to *Locusta migratoria* and *Eyprepocnemis monticola* to *Eumigus monticola* in the following paragraph:

“With the RepeatExplorer and RepeatMasker programs, the satellite DNA composition of the migratory locust (*Locusta migratoria*) B chromosomes was determined [19]. The ‘satellitome’ in the grasshopper *Eumigus monticola* consists of 27 satellite DNAs [6], less than half of the migratory locust, where 62 were found [19].”

The changes do not affect the scientific results. The manuscript will be updated, and the original will remain online on the article webpage, with a reference to this Correction.
